# A novel prohibitin-binding compound induces the mitochondrial apoptotic pathway through NOXA and BIM upregulation

**DOI:** 10.18632/oncotarget.6154

**Published:** 2015-10-19

**Authors:** Cristina Moncunill-Massaguer, José Saura-Esteller, Alba Pérez-Perarnau, Claudia Mariela Palmeri, Sonia Núñez-Vázquez, Ana M. Cosialls, Diana M. González-Gironès, Helena Pomares, Anne Korwitz, Sara Preciado, Fernando Albericio, Rodolfo Lavilla, Gabriel Pons, Thomas Langer, Daniel Iglesias-Serret, Joan Gil

**Affiliations:** ^1^ Departament de Ciències Fisiològiques II, Universitat de Barcelona-Institut d'Investigació Biomèdica de Bellvitge (IDIBELL), L'Hospitalet de Llobregat, Catalunya, Spain; ^2^ Institute for Genetics and Cologne Excellence Cluster on Cellular Stress Responses in Aging-Associated Diseases (CECAD), University of Cologne, Cologne, Germany; ^3^ Barcelona Science Park and CIBER-BBN, Networking Centre on Bioengineering, Biomaterials and Nanomedicine, Barcelona, Spain; ^4^ Institute for Research in Biomedicine Barcelona, Barcelona, Spain; ^5^ Department of Organic Chemistry, University of Barcelona, Barcelona, Spain; ^6^ Laboratory of Organic Chemistry, Faculty of Pharmacy, University of Barcelona, Barcelona, Spain

**Keywords:** apoptosis, prohibitins, BCL-2 family members, mitochondria, cancer

## Abstract

We previously described diaryl trifluorothiazoline compound 1a (hereafter referred to as fluorizoline) as a first-in-class small molecule that induces p53-independent apoptosis in a wide range of tumor cell lines. Fluorizoline directly binds to prohibitin 1 and 2 (PHBs), two proteins involved in the regulation of several cellular processes, including apoptosis. Here we demonstrate that fluorizoline-induced apoptosis is mediated by PHBs, as cells depleted of these proteins are highly resistant to fluorizoline treatment. In addition, BAX and BAK are necessary for fluorizoline-induced cytotoxic effects, thereby proving that apoptosis occurs through the intrinsic pathway. Expression analysis revealed that fluorizoline induced the upregulation of *Noxa* and *Bim* mRNA levels, which was not observed in PHB-depleted MEFs. Finally, *Noxa*^−/−^/*Bim*^−/−^ MEFs and *NOXA-*downregulated HeLa cells were resistant to fluorizoline-induced apoptosis. All together, these findings show that fluorizoline requires PHBs to execute the mitochondrial apoptotic pathway.

## INTRODUCTION

**Figure 1:**
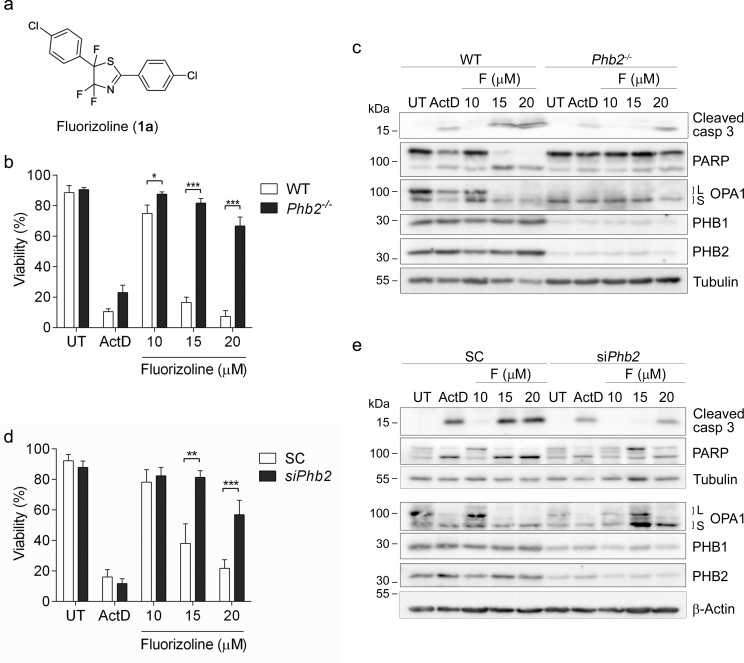
The presence of PHBs is required for fluorizoline-induced apoptosis **a.** Chemical structure of fluorizoline (diaryl trifluorothiazoline compound 1a). **b.**, **c.** Cre recombinase was transduced in WT and *Phb2^fl/fl^* (*Phb2^−/−^*) MEFs for 72 h. Then cells were untreated (UT) or treated with either 0.15 μg/mL Actinomycin D (ActD) or increasing doses of fluorizoline (F) for 24 h. **d.**, **e.** *Phb2^fl/fl^* MEFs were transfected with scramble (SC) or *Phb2* (si*Phb2*) siRNA for 72 h. Afterwards, cells were treated with either 0.15 μg/mL Actinomycin D (ActD) or increasing doses of fluorizoline (F) for 24 h. **b.**, **d.** Viability was measured by flow cytometry and it is expressed as the mean ± SEM (*n* ≥ 3) of the percentage of non-apoptotic cells (annexin V-negative). *p < 0.05, **p < 0.01, ***p < 0.001. **c.**, **e.** Protein levels were analyzed by western blot. Tubulin and β-Actin were used as a loading control. These are representative images of at least three independent experiments.

Resistance to cell death is one of the hallmarks of cancer development, and it compromises the efficacy of conventional anti-cancer agents [1]. Hence, there is need for the development of novel apoptosis-inducing compounds as potential therapies for cancer. In a previous report we described the synthesis of a class of small molecules with an unprecedented fluorinated thiazoline scaffold as novel pro-apoptotic compounds [2]. Interestingly, the diaryl trifluorothiazoline compound 1a (Figure 1a), hereafter referred to as fluorizoline, triggered apoptosis through a p53-independent mechanism, which is highly relevant as half of all cancers acquire mutations in p53 during the malignant transformation [3]. In addition, we identified prohibitin 1 and 2 (PHBs) as proteins selectively binding to fluorizoline [2].

PHBs are evolutionary conserved, ubiquitously expressed proteins that are required for cellular proliferation, development, and the functional integrity of mitochondria [4-6]. Consistently, depletion of PHBs leads to an impairment in embryonic development in *Caenorhabditis elegans* and mice [7-10]. PHBs are predominantly found in mitochondria, but they have also been identified in other cell compartments, including the plasma membrane and the nucleus [4, 11]. Within the inner mitochondrial membrane (IMM), PHB1 and PHB2 interact with each other to form large heteromeric ring-like complexes, which presumably serve as membrane scaffolds [6, 12]. Cells depleted of PHBs show fragmented mitochondria with an altered ultrastructure, as well as marked degradation of L-OPA1 [10, 13], a mitochondrial dynamin-like GTPase that mediates mitochondrial fusion and maintains cristae structure [14, 15]. In addition, PHBs have been linked to oxidative stress, as their absence induces the production of reactive oxygen species [16-19], and their overexpression protects against oxidative stress [18, 20, 21]. Loss of PHBs leads to impaired proliferation [10, 13, 22] and increased sensitivity to apoptotic stimuli [10, 11, 18, 23-25]. Thus, PHBs play a key role in the maintenance of the functional integrity of mitochondria, which ultimately allows proper cell and tissue homeostasis [4,6,26-28].

The role of PHBs in cancer remains controversial [11, 29]. An anti-tumorigenic role for PHBs was reported in various tumor cell types [30-33]. In contrast, PHBs have been linked to tumor growth, resistance to chemotherapy and metastasis [11, 29, 34]. The requirement of PHBs for cell proliferation strongly indicates a potential role in cancer progression [4, 11, 12, 22, 35]. Along this line, overexpression of PHB1 results in a higher resistance to apoptosis in different types of cancer cells, while downregulation of PHBs renders cancer cells more susceptible to pro-apoptotic insults [11, 21, 29, 36], suggesting a pro-tumorigenic role of PHBs.

In view of the pleiotropic functions of PHBs, these proteins emerge as highly interesting targets for the development of novel treatments for cancer [11, 29, 37]. In this regard, we propose that PHB-binding fluorizoline is a promising new agent due to its pro-apoptotic capacity.

Here we further dissect the mechanism by which fluorizoline induces apoptosis and demonstrate that this compound requires PHBs to increase *Noxa* and *Bim* expression and to trigger the mitochondrial apoptotic pathway.

## RESULTS

### Fluorizoline requires the presence of prohibitins in order to induce apoptosis

We previously reported that fluorizoline (Figure 1a) binds specifically to PHB1 and PHB2 [2]. To examine whether the pro-apoptotic effects of this compound require PHBs, we sought to analyze the response of PHB-deficient cells to fluorizoline treatment. To this end, we used *Phb2^fl/fl^* MEFs, in which Cre-recombinase transduction induces loss of the *Phb2* gene [10]. PHBs are interdependent at the protein level, thus loss of PHB2 leads to the degradation of PHB1 [9, 10]. As a control, we used wild type (WT) MEFs, in which Cre-recombinase transduction does not modify PHB protein levels. Seventy-two hours after Cre-recombinase transduction, WT and *Phb2^fl/fl^* MEFs were treated with increasing doses of fluorizoline for 24 h, and cell viability was analyzed by flow cytometry. While WT MEFs were sensitive to fluorizoline treatment, loss of PHBs conferred strong resistance to fluorizoline-induced apoptosis in Cre-transduced *Phb2^fl/fl^* MEFs (*Phb2*^−/−^ MEFs) (Figure 1b). In contrast, both cell lines showed a similar response to Actinomycin D (ActD). Western blot analysis revealed that Cre-mediated depletion of *Phb2* was accompanied by loss of PHB1 and L-OPA1 (Figure 1c), as previously described [10]. In addition, fluorizoline treatment led to cleavage of caspase 3 and poly ADP-ribose polymerase (PARP) in WT MEFs, thereby indicating induction of apoptosis. In contrast, the cleavage of these two proteins was clearly reduced in fluorizoline-treated *Phb2*^−/−^ MEFs (Figure 1c). Contrary to fluorizoline, ActD triggered similar cleavage of caspase 3 and PARP in WT and *Phb2*^−/−^ MEFs. Furthermore, disappearance of L-OPA1 forms was observed in fluorizoline- and ActD- treated WT MEFs.

Treatment with fluorizoline triggered apoptosis in WT and *Phb2^fl/fl^* MEFs in a similar manner in the absence of Cre-recombinase (Supplementary Figure 1a). Accordingly, fluorizoline and ActD induced a similar processing of caspase 3, PARP, and L-OPA1 in WT and *Phb2^fl/fl^* MEFs (Supplementary Figure 1b). These findings demonstrate that the resistance observed in *Phb2^−/−^* MEFs was not due to intrinsic insensitivity to apoptotic insults of the cell line but to the loss of PHBs.

In order to corroborate that lack of PHBs confers resistance to fluorizoline-induced apoptosis, *Phb2* levels were downregulated by siRNA transfection in *Phb2^fl/fl^* MEFs. Reduction of *Phb2* resulted in decreases of PHB1 and L-OPA1 (Figure 1e). As expected, the pro-apoptotic effects of fluorizoline were blocked when PHBs were downregulated, as shown by flow cytometry and western blot analysis (Figure 1d-1e). These results were further confirmed by *Phb2* siRNA transfection in WT MEFs (Supplementary Figure 1c-d). All together, these findings demonstrate that PHBs are necessary for fluorizoline-induced apoptosis.

Furthermore, we sought to analyze whether modulation of PHB expression levels affects the sensitivity to fluorizoline. To this end, PHBs were overexpressed in *Phb2^fl/fl^* MEFs and the response to fluorizoline was examined. Interestingly, fluorizoline efficiently triggered apoptosis in spite of PHB overexpression (Supplementary Figure 2a-b). Similar results were obtained by overexpressing PHBs in HeLa cells (Supplementary Figure 2c-d).

In addition, the pro-apoptotic effects of fluorizoline were analyzed in *Phb2^fl/−^* MEFs, which show lower levels of PHBs compared to *Phb2^fl/fl^* (Supplementary Figure 3a). Cells were incubated with fluorizoline for 24 h and viability was analyzed by flow cytometry. Strikingly, *Phb2^fl/−^* and *Phb2^fl/fl^* clones displayed similar sensitivity to fluorizoline treatment (Supplementary Figure 3b). Taken together, these results show that the protein levels of PHBs do not strictly correlate with the sensitivity to fluorizoline. Resistance to fluorizoline can only be observed in the absence of PHBs.

### Fluorizoline induces mitochondrial fragmentation and cristae disorganization

**Figure 2:**
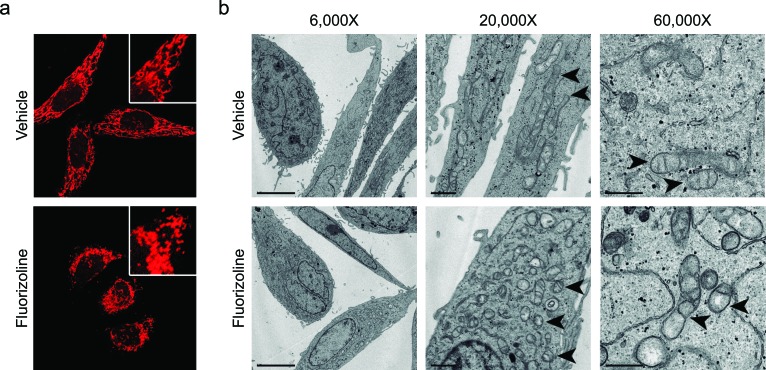
Treatment with fluorizoline induces changes in the mitochondrial morphology and ultrastructure **a.** HeLa cells were treated with DMSO (vehicle) or 10 μM fluorizoline for 4 h. Mitochondria were stained with 100 nM MitoTracker^®^ Red CMXRos and imaged using a confocal microscope. **b.** HeLa cells were incubated with DMSO (vehicle) or 2 μM fluorizoline for 24 h and changes in the mitochondrial morphology were visualized by transmission electron microscopy. Magnification at 6,000X (scale corresponding to 5 μm), 20,000X (scale corresponding to 1 μm), and 60,000X (scale corresponding to 0.5 μm). Arrowheads indicate the mitochondria.

Upon apoptosis induction with various stimuli, the mitochondrial network undergoes extensive fragmentation, along with cristae remodeling [38-41]. Interestingly, this phenotype was also observed upon loss of PHBs, which was attributed to the accelerated processing of L-OPA1 [10, 13]. We sought to analyze mitochondrial morphology and ultrastructure after fluorizoline treatment. HeLa cells incubated with fluorizoline presented fragmented mitochondria that clustered around the nucleus (Figure 2a), as well as disarrangements of cristae, which appeared dilated and disorganized (Figure 2b). Higher magnification micrographs (60,000X) showed that incubation with fluorizoline induced partial or total loss of cristae, along with remodeling of the IMM into many separate vesicular and electron-lucent matrix compartments (Figure 2b). Therefore, these results show that fluorizoline treatment leads to mitochondrial fragmentation and cristae disorganization. These findings are consistent with the capacity of fluorizoline to induce apoptosis and with PHBs being the target of this compound.

To analyze the role of OPA1 in fluorizoline-induced apoptosis, we overexpressed variant 1 of OPA1 lacking the S1 cleavage site (OPA1-ΔS1), a non-cleavable long form of OPA1 [42]. Strikingly, fluorizoline could efficiently induce apoptosis in the presence of OPA1-ΔS1 (Supplementary Figure 4a-b), suggesting that the loss of L-OPA1 is not required for the pro-apoptotic effects of fluorizoline. To confirm these results, we used MEFs lacking OMA1, a mitochondrial protease that cleaves OPA1 in response to several stress insults [43-47]. Fluorizoline treatment triggered the loss of L-OPA1 in WT MEFs, while *Oma1^−/−^* MEFs maintained L-OPA1 forms even in the presence of fluorizoline. In contrast, WT and *Oma1^−/−^* MEFs showed a similar response to fluorizoline (Supplementary Figure 4c-d). All together, these results prove that fluorizoline does not rely on stress-induced loss of L-OPA1 forms to induce apoptosis.

### Fluorizoline increases the production of reactive oxygen species independently of PHBs and apoptosis induction

We analyzed the effects of fluorizoline on the mitochondrial membrane potential (ΔΨm) as an indicator of mitochondrial function. As apoptosis induction can affect ΔΨm [39], it was important to analyze ΔΨm before the onset of cell death. To this end, we used Jurkat cells, as previous results from our group showed that cytochrome *c* was released from mitochondria after 8 h of fluorizoline treatment [2]. Interestingly, 8 h of fluorizoline treatment resulted only in a slight loss of ΔΨm (Supplementary Figure 5), which suggests that it is a consequence of apoptosis induction.

**Figure 3:**
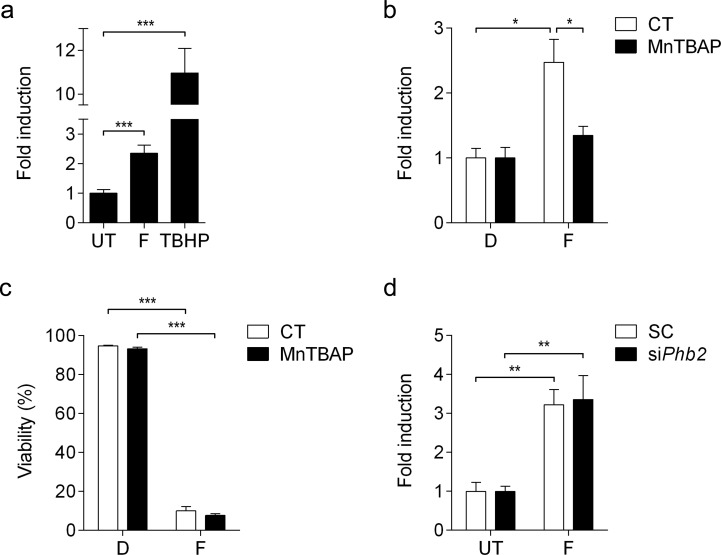
Fluorizoline induces ROS production independently of PHBs and apoptosis induction **a.** Jurkat cells were untreated (UT), or treated with 10 μM fluorizoline (F) for 1 h or 200 μM tert-butyl hydroperoxide (TBHP) for 30 min. **b.** Jurkat cells were untreated (CT) or pre-incubated with 150 μM MnTBAP for 1 h and then treated with DMSO (D) or 10 μM fluorizoline (F) for 1 h. **c.** Jurkat cells were untreated (CT) or pre-incubated with 150 μM MnTBAP for 1 h and then treated with DMSO (D) or 10 μM fluorizoline (F) for 24 h. Viability was measured by flow cytometry and it is expressed as the mean ± SEM (*n* = 3) of the percentage of non-apoptotic cells (annexin V-negative). **d.** *Phb2^fl/fl^* MEFs were transfected with scramble (SC) or *Phb2* (si*Phb2*) siRNA for 72 h. Cells were then untreated or treated with 20 μM fluorizoline (F) for 1 hour. **a., b., d.** Superoxide anion and hydroxyl radical production was analyzed by flow cytometry using CellROX^®^ Deep Red Reagent. Data show the mean values ± SEM relative to the mean of the control (a, *n* = 9; b, *n* = 3; d, *n* = 4). *p < 0.05, **p < 0.01, ***p < 0.001.

In addition, we sought to analyze whether treatment with fluorizoline leads to generation of reactive oxygen species (ROS) by using CellROX^®^ Deep Red reagent, which detects superoxide anion and hydroxyl radical. Jurkat cells showed increased ROS production upon treatment with fluorizoline for 1 h (Figure 3a). In order to study the involvement of ROS in fluorizoline-induced apoptosis, we pre-treated cells with the SOD mimetic manganese (III)-tetrakis (4-benzoic acid) porphyrin (MnTBAP), which efficiently inhibited fluorizoline-induced ROS production (Figure 1d), while it could not revert the apoptotic effects of fluorizoline (Figure 3c). Furthermore, we analyzed the production of ROS in *Phb2*-downregulated MEFs, which were resistant to fluorizoline-induced apoptosis (Figure 1a). Importantly, the absence of PHBs could not inhibit the generation of ROS induced by fluorizoline (Figure 3d). All together, these results demonstrate that fluorizoline does not require the generation of ROS to induce apoptosis.

### Fluorizoline induces the mitochondrial apoptotic pathway in a BAX/BAK-dependent manner

**Figure 4:**
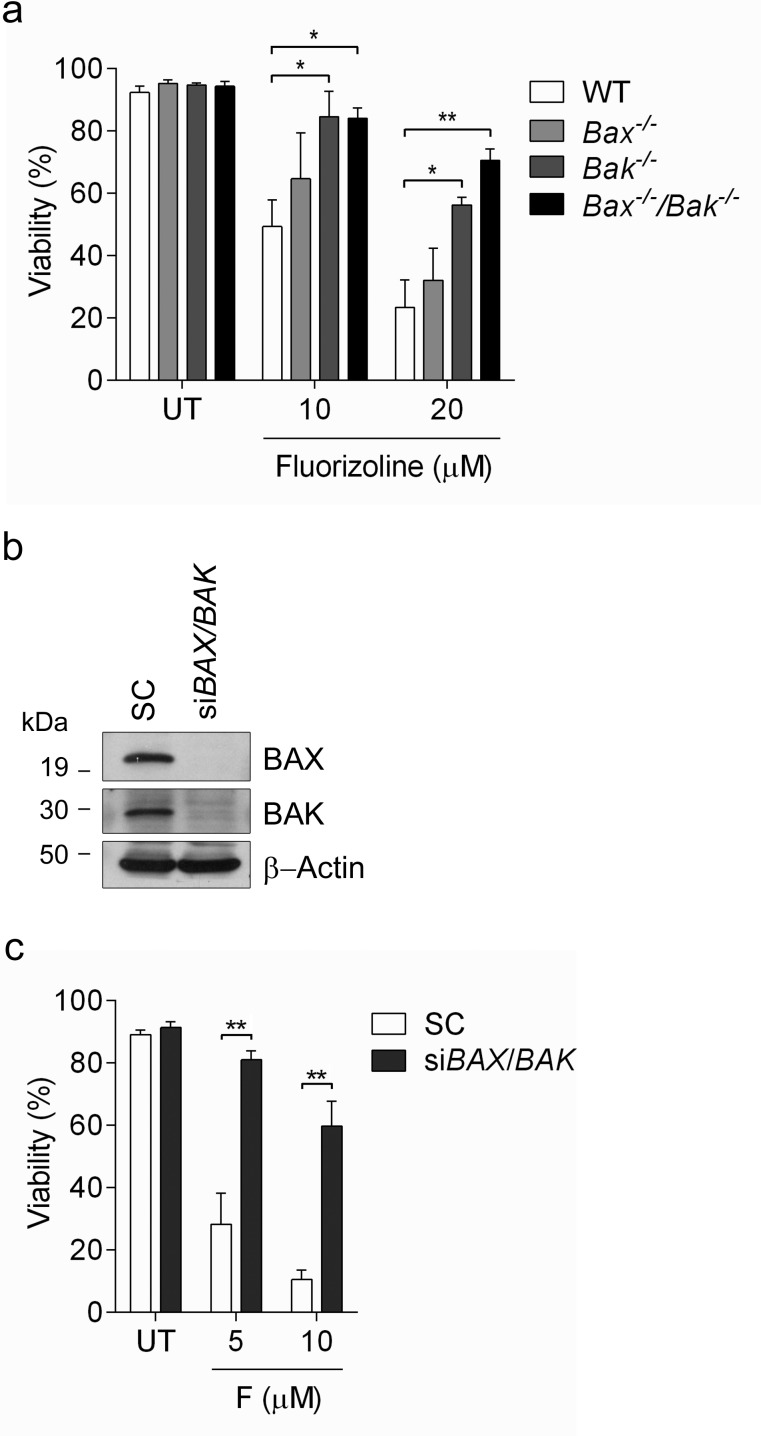
Fluorizoline-induced apoptosis occurs through the mitochondrial pathway in a BAX- and BAK-dependent manner **a.** WT, *Bax*^−/−^, *Bak*^−/−^ or *Bax*^−/−^/*Bak*^−/−^ MEFs were untreated (UT) or treated with 10 or 20 μM fluorizoline for 24 h. **b.**, **c.** HeLa cells were transfected with scramble (SC) or *BAX* and *BAK* siRNA (si*BAX*/*BAK*) for 48 h. **b.** The efficiency of gene silencing was validated by western blot. β-Actin was used as a loading control. This is a representative image of at least three independent experiments. **c.** HeLa cells were untreated (UT) or treated with 5 or 10 μM fluorizoline (F) for 24 h. **a.**, **c.** Viability was measured by flow cytometry and it is expressed as the mean ± SEM (n≥3) of the percentage of non-apoptotic cells (annexin V-negative).*p < 0.05, **p < 0.01.

The execution of apoptosis depends on the balance between pro- and anti-apoptotic BCL-2 family members [48, 49]. BAX and BAK are two multidomain pro-apoptotic proteins whose activation leads to mitochondrial outer membrane permeabilization (MOMP) and the release of mitochondrial intermembrane proteins such as cytochrome *c*, two critical steps in the execution of apoptosis [48, 49]. In order to evaluate the role of BAX and BAK in fluorizoline-induced apoptosis, we used *Bax*^−/−^, *Bak*^−/−^ and *Bax*^−/−^/*Bak*^−/−^ MEFs, which are resistant to multiple cell death stimuli that induce apoptosis through MOMP [50]. Importantly, fluorizoline triggered apoptosis in WT MEFs, while the absence of both BAX and BAK significantly prevented the cytotoxic effects of this compound (Figure 4a). *Bax*^−/−^ MEFs were as sensitive to fluorizoline as WT MEFs, although *Bak*^−/−^ MEFs offered some resistance to the apoptosis induced by this compound.

To corroborate these results, HeLa cells were transiently depleted of these two pro-apoptotic proteins by siRNA transfection (Figure 4b). Consistently, depletion of BAX and BAK reduced the apoptotic response to fluorizoline treatment (Figure 4c).

To confirm the importance of the activation of the intrinsic apoptotic pathway, we used Jurkat cells overexpressing BCL-X_L_ [51]. This is an anti-apoptotic member of the BCL-2 family, whose overexpression can block the intrinsic pathway of apoptosis [51, 52]. Fluorizoline was able to induce apoptosis in WT Jurkat cells, while overexpression of BCL-X_L_ conferred resistance to the pro-apoptotic effects of fluorizoline (Supplementary Figure 6a-b).

Although the lack of BAX and BAK conferred significant resistance to fluorizoline treatment, cell viability was still slightly decreased. Pre-treatment of *Bax*^−/−^/*Bak*^−/−^ MEFs with the pan-caspase inhibitor Q-VD-OPh completely blocked fluorizoline-induced apoptosis (Supplementary Figure 6c). As the intrinsic apoptotic pathway is blocked in the absence of BAX and BAK, we assessed the role of the extrinsic apoptotic pathway by adding the caspase 8 inhibitor Z-IETD-FMK. Strikingly, inhibiting caspase 8 activity did not have any effect on fluorizoline-induced apoptosis in WT MEFs, while it could efficiently prevent the pro-apoptotic effects of fluorizoline in *Bax*^−/−^/*Bak*^−/−^ MEFs (Supplementary Figure 6c). To corroborate these results, we used *Bax*^−/−^/*Bak*^−/−^ MEFs overexpressing CrmA, a cowpox virus protein that preferentially inhibits caspase 8 [53]. Consistently, CrmA effectively inhibited apoptosis induced by fluorizoline in *Bax*^−/−^/*Bak*^−/−^ MEFs (Supplementary Figure 6d). All together, these findings show that the mitochondrial apoptotic pathway is essential for fluorizoline-induced apoptosis. Strikingly, in the absence of BAX and BAK, fluorizoline induces a minor decrease in viability by activating caspase 8.

### Prohibitins mediate the modulation of the expression of various BCL-2 family members upon fluorizoline treatment

**Figure 5:**
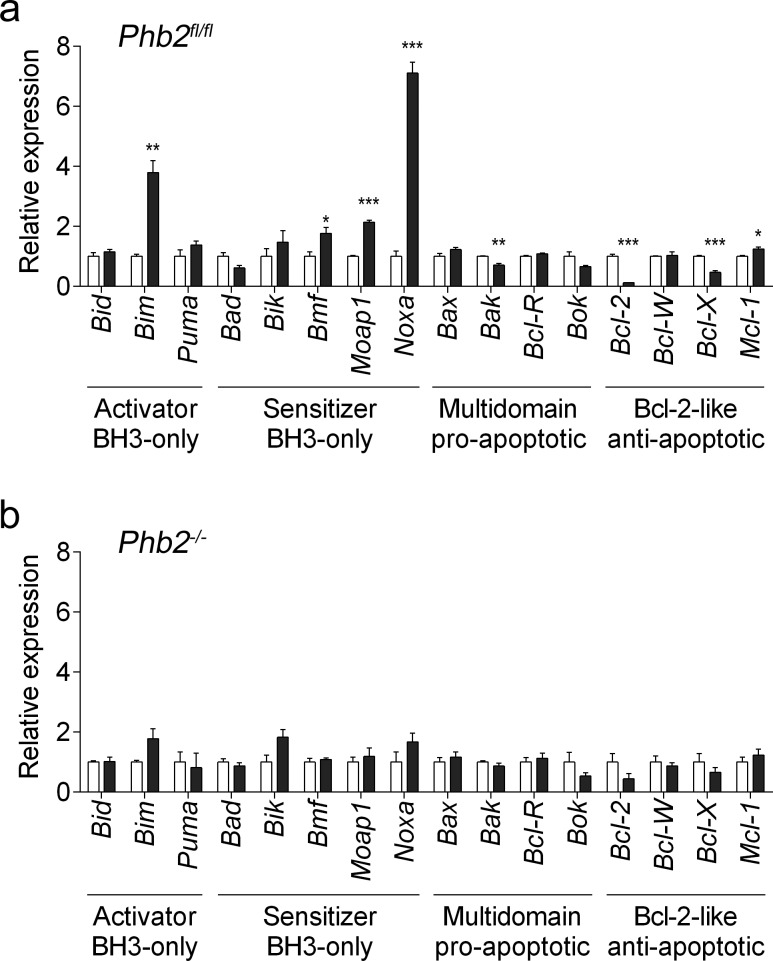
Fluorizoline modulates the expression of BCL-2 family members by targeting PHBs in MEFs **a.** *Phb2^fl/fl^* MEFs were untreated (white bars) or treated with 20 μM fluorizoline (black bars) for 24 h. **b.** *Phb2^fl/fl^* MEFs were transduced with Cre recombinase for 72 h (*Phb2^−/−^*) and then untreated or treated with 20 μM fluorizoline for 24 h. **a.**, **b.** mRNA levels were analyzed by RT-MLPA. White bars correspond to untreated cells, and black bars to fluorizoline-treated cells. Data show the mean values ± SEM of three independent experiments relative to the mean of the control. *p < 0.05, **p < 0.01, ***p < 0.001 untreated versus treated cells.

To gain insight into the mechanism of apoptosis induction upon fluorizoline treatment, changes in the overall apoptosis mRNA expression profile were analyzed by reverse transcriptase multiplex ligation-dependent probe amplification (RT-MLPA) in *Phb2^fl/fl^* MEFs. After 24 h of treatment with 20 μM fluorizoline, the upregulation of the pro-apoptotic genes *Bim*, *Noxa*, *Bmf*, and *Moap1* was observed, along with downregulation of the anti-apoptotic genes *Bcl-2* and *Bcl-X* (Figure 5a and Supplementary Figure 7a).

Fluorizoline-induced apoptosis requires the presence of PHBs (Figure 1). It is therefore conceivable that the modulations observed in apoptosis-related gene expression do not occur in cells lacking PHBs. To test this hypothesis, we transduced *Phb2^fl/fl^* MEFs with Cre-recombinase for 72 h and then treated them with 20 μM fluorizoline for 24 h. Importantly, Cre-mediated loss of PHBs abolished the modulations of mRNA levels triggered by fluorizoline (Figure 5b and Supplementary Figure 7b). Thus, the presence of PHBs is required for fluorizoline-induced changes in the apoptosis-related mRNA expression profile.

Interestingly, treatment with fluorizoline increased BIM protein levels in *Phb2^fl/fl^* MEFs, although these levels were not modulated in *Phb2^−/−^* MEFs (Supplementary Figure 8). NOXA protein levels could not be analyzed due to lack of proper antibodies with the capacity to detect the mouse protein (data not shown). Consequently, we further studied fluorizoline-induced apoptosis by analyzing changes in the expression of BCL-2 family members in HeLa cells, as there are more antibodies available for the detection of human BCL-2 family proteins.

**Figure 6:**
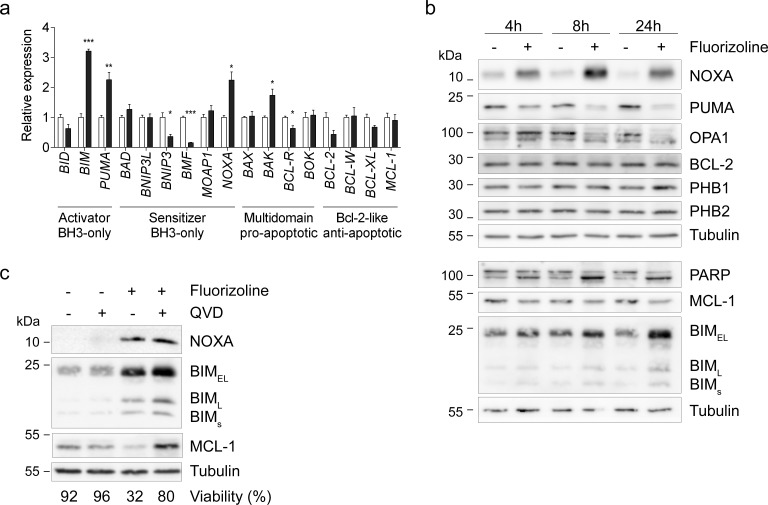
Fluorizoline changes mRNA and protein levels of various BCL-2 family members in HeLa cells **a.** HeLa cells were untreated (white bars) or treated with 10 μM fluorizoline (black bars) for 24 h. mRNA levels were analyzed by RT-MLPA. Data show the mean values ± SEM of three independent experiments relative to the mean of the control. *p < 0.05, **p < 0.01, ***p < 0.001 untreated versus treated cells. **b.** HeLa cells were untreated or treated with 10 μM fluorizoline for 4, 8 and 24 h. **c.** HeLa cells were pre-incubated with 20 μM caspase inhibitor Q-VD-OPh for 30 min and then treated with 10 μM fluorizoline for 24 h. **b.**, **c.** Protein levels were analyzed by western blot. Tubulin was used as a loading control. These are representative images of at least three independent experiments.

First, the apoptosis-related gene expression profile was examined by RT-MLPA in HeLa cells. Treatment with 10 μM fluorizoline increased the pro-apoptotic *BIM*, *PUMA, NOXA*, and *BAK* mRNA levels (Figure 6a and Supplementary Figure 9). Next, we sought to examine whether these modulations of the mRNA levels resulted in changes in the corresponding protein levels. We observed a time-dependent upregulation of NOXA and BIM, as well as decreases in PUMA and MCL-1 protein levels (Figure 6b). Despite the decreases in *BCL-2* mRNA levels, fluorizoline did not significantly modulate its protein levels, a finding consistent with the long half-life of this protein [54]. In addition, PHB protein levels were not modified upon fluorizoline treatment. Interestingly, increases in NOXA and BIM preceded caspase activation, as pre-incubation with pan-caspase inhibitor Q-VD-OPh did not block their upregulation (Figure 6c). In contrast, caspase inhibition abolished the decrease in MCL-1 mediated by fluorizoline treatment, thereby demonstrating that it is a caspase-dependent event. Thus, PHBs mediate fluorizoline-induced changes in *Noxa* and *Bim* expression, the protein levels of which increased prior to caspase activation. These modulations could explain the apoptotic outcome observed.

To validate the increases in BIM and NOXA protein levels, we analyzed the effects of fluorizoline in primary cancer cells. Specifically, we used cells from four patients with different hematological malignancies, namely chronic myeloid leukemia-derived blast crisis, mantle cell lymphoma, B cell chronic lymphoproliferative syndrome and adult T-cell leukemia/lymphoma. Interestingly, treatment with fluorizoline resulted in a decrease in viability and an increase in NOXA protein levels, whereas BIM protein levels were not modified (Supplementary Figure 10a-d). We also analyzed changes in BCL-2 family members following fluorizoline treatment in various cancer cell lines. NOXA and BIM protein levels increased in a dose-dependent manner in HT-29, a colon cancer cell line, as well as in A375P and WM1552, two melanoma cell lines (Supplementary Figure 10e-f). In Jurkat cells, we only observed fluorizoline-induced increases in NOXA protein levels (Supplementary Figure 10g). Hence, fluorizoline seems to mainly increase NOXA protein levels, while BIM is also upregulated in some cancer cells.

### Role of NOXA and BIM in fluorizoline-induced apoptosis

**Figure 7:**
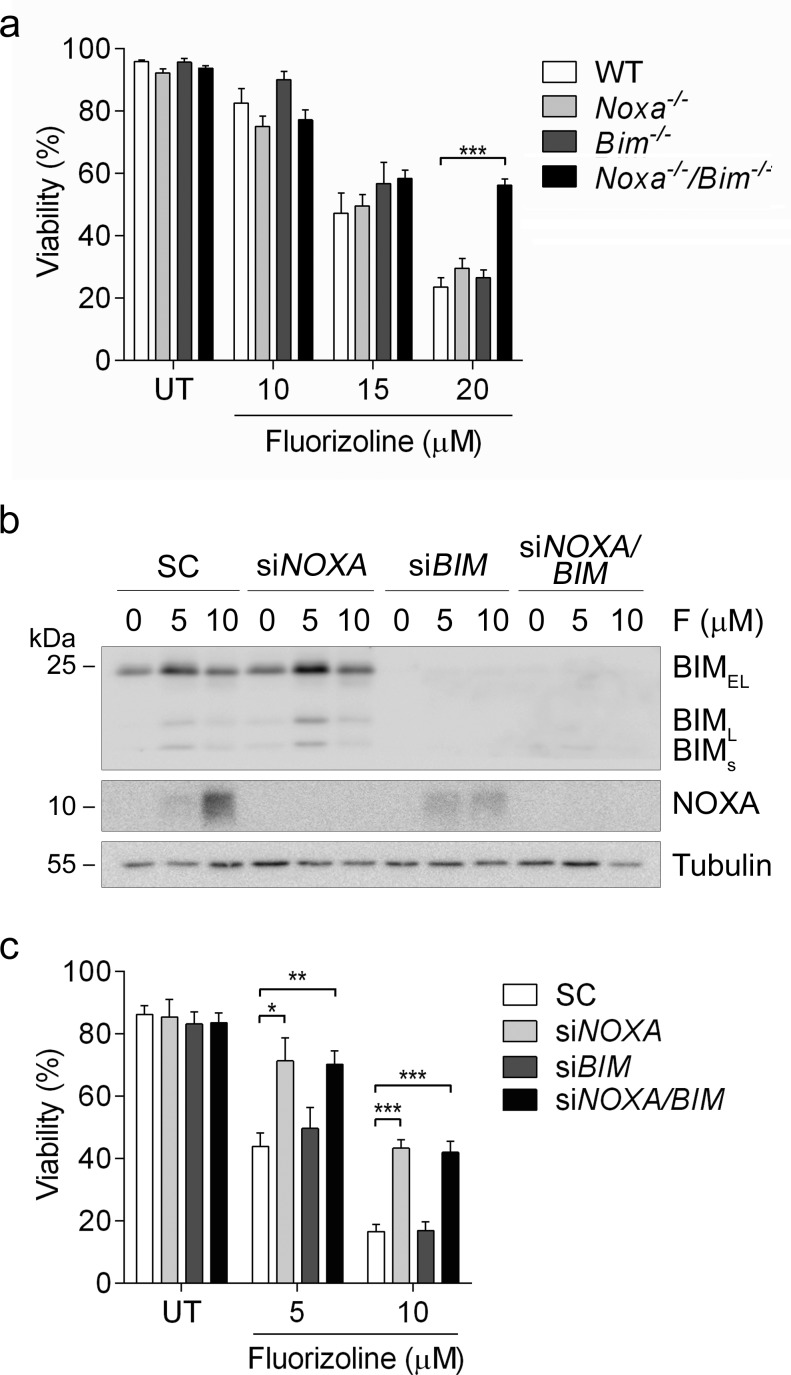
Loss of NOXA and BIM blocks fluorizoline-induced apoptosis **a.** WT, *Noxa*^−/−^, *Bim*^−/−^ or *Noxa*^−/−^/*Bim*^−/−^ MEFs were untreated (UT) or treated with 10, 15 or 20 μM of fluorizoline for 24 h. **b., c.** HeLa cells were transfected with scramble (SC), *NOXA* siRNA (si*NOXA*), *BIM* siRNA (si*BIM*), or both *NOXA* and *BIM* siRNA (si*NOXA*/*BIM*) for 48 h and then untreated (UT) or treated with 5 or 10 μM fluorizoline (F) for 24 h. **b.** Protein levels were analyzed by western blot. Tubulin was used as a loading control. This is a representative image of at least three independent experiments. **a.**, **c.** Viability was measured by flow cytometry and it is expressed as the mean ± SEM (*n* ≥ 3) of the percentage of non-apoptotic cells (annexin V-negative).*p < 0.05, **p < 0.01, ***p < 0.001.

We sought to further study the role of NOXA and BIM in fluorizoline-induced apoptosis. To this end, we performed a viability assay in MEFs lacking *Noxa*, *Bim*, or both. *Noxa*^−/−^/*Bim*^−/−^ MEFs were resistant to the pro-apoptotic effects of fluorizoline, whereas WT, *Noxa*^−/−^ and *Bim*^−/−^ MEFs showed a similar sensitivity to this compound (Figure 7a). In addition, *NOXA* and *BIM* were downregulated by siRNA transfection in HeLa cells and their response to fluorizoline treatment was assessed. Cells lacking BIM were sensitive to the pro-apoptotic effects of fluorizoline, while lack of NOXA conferred resistance to these effects. Simultaneous depletion of NOXA and BIM did not increase resistance to this compound when compared to depletion of NOXA alone (Figure 7b-7c).

All together, these data demonstrate that fluorizoline induces the mitochondrial apoptotic pathway predominantly through the upregulation of NOXA and, depending on the cell type, also of BIM.

## DISCUSSION

In the present study we describe the mechanism of apoptosis induction exerted by fluorizoline, a new pro-apoptotic agent with a fluorinated thiazoline scaffold. It was previously described that fluorizoline directly binds to PHB1 and PHB2 [2]. Here we show that PHBs mediate the pro-apoptotic effects of fluorizoline through two approaches. Depletion of *Phb2* either by Cre-mediated recombination in *Phb2^fl/fl^* MEFs or by siRNA transfection in MEFs conferred resistance to fluorizoline. Hence, fluorizoline is a new synthetic agent that reduces cell viability by specifically targeting PHBs.

PHBs play a key role in the maintenance of mitochondrial homeostasis. High expression of PHBs has been found in cells that heavily depend on mitochondrial function, notably proliferating cells. It is conceivable that these cells are particularly susceptible to the lack of functional PHBs [4]. In fact, downregulation of PHBs in various cell lines blocks cell proliferation [10, 22, 35] and also induces apoptosis [13, 55, 56]. Overexpression of PHBs protects cells from apoptosis induced by various stimuli, an observation that is consistent with PHB1 having a cytoprotective role [11]. In MEFs, PHBs exert their anti-apoptotic function via L-OPA1, as the increased sensitivity to apoptosis observed in the absence of PHBs can be counteracted by re-expression of a non-cleavable variant of L-OPA1 [10]. In addition, it has been reported that PHB overexpression inhibits apoptosis through the induction of BCL-2 and BCL-X_L_ [23] and that downregulation of PHBs reduces BCL-X_L_ levels [57]. Interestingly, overexpression of BCL-X_L_ protected cells from fluorizoline-induced apoptosis.

The role of PHBs in cancer has been largely discussed. Although some reports point to an anti-tumorigenic role of PHBs, a growing body of evidence links these proteins to tumor development [11, 21, 29, 34, 35]. Fluorizoline has the capacity to induce apoptosis in a wide range of cancer cell lines, including glioma and prostate cancer cell lines [2], in which PHBs were reported to have an anti-tumorigenic role [31, 32]. Thus, the induction of apoptosis by targeting PHBs emerges as a new therapy for cancer.

The exact mechanism by which fluorizoline induces apoptosis remains to be elucidated. The loss of either PHB1 or PHB2 may destabilize mitochondrial PHBs complexes, leading to the activation of quality control mechanisms, which would in turn degrade the other partner [6, 58]. Therefore, the observation that PHB protein levels are maintained upon fluorizoline treatment suggests that the PHBs ring-like complex in the IMM is not destabilized. Treatment with fluorizoline increased the production of ROS, as observed in the absence of PHBs in various cell types [16-19, 24, 59, 60]. However, we proved that the generation of ROS was independent of PHBs and apoptosis induction, discarding oxidative stress as a mechanism of action of fluorizoline. PHBs are known to physically interact with several subunits of complex I and II of the respiratory chain, presumably contributing to their proper assembly and stability [61-63]. In fact, deletion of PHBs decreases the activity of various complexes of the respiratory chain [16, 26, 59]. We cannot exclude that fluorizoline will affect the activity of these complexes due to its PHBs-binding properties.

Treatment with fluorizoline induces fragmentation of the mitochondrial network, cristae remodeling and loss of L-OPA1, which is consistent with the role of this protein in mitochondrial fusion and in the maintenance of cristae structure [14, 15]. Strikingly, PHB-depleted cells also present fragmented mitochondrial network and aberrant cristae morphogenesis, which are attributed to the loss of L-OPA1 [10]. However, it is worth mentioning that this observation cannot be exclusively interpreted as a proof that fluorizoline targets PHBs. Other apoptotic stimuli have been reported to trigger the same changes in mitochondrial structure [38-41] and the processing of L-OPA1 [42, 64, 65], which allows reorganization of mitochondrial cristae and facilitates cytochrome *c* release [66, 67]. Our results demonstrate that fluorizoline was able to induce apoptosis independently of the loss of L-OPA1 forms, which excludes this event as the cause of apoptosis induction.

We have shown that fluorizoline triggers the intrinsic apoptotic pathway, as BAX and BAK are required for fluorizoline-induced apoptosis. The protection observed in *Bak*^−/−^ MEFs could be related to the recently reported requirement of BAK for efficient BAX translocation and oligomerization at the outer mitochondrial membrane [68]. The activation of the intrinsic apoptotic pathway involving BCL-2 family members appears to be necessary for the successful outcome of many cancer treatments [48]. Expression analysis revealed a consistent upregulation of two BH3-only proteins, namely the activator *Bim* and the sensitizer *Noxa* in MEF and HeLa cells upon fluorizoline treatment. Of note, these modulations were dependent on the presence of PHBs in MEFs.

Importantly, fluorizoline induced increases in NOXA and BIM protein levels prior to caspase activation, which could explain the apoptotic outcome. NOXA and BIM are essential for the induction of apoptosis in response to a variety of pro-apoptotic insults [48, 69-71]. The lack of NOXA in HeLa cells conferred resistance to the pro-apoptotic effects of fluorizoline, although this was not observed in *Noxa^−/−^* MEFs. These observations are consistent with the reported differences in the apoptotic potential of NOXA in function of the cell type [70]. Specifically, NOXA overexpression induces significant apoptosis in HeLa cells [72], while in MEFs it is poorly apoptotic [73, 74]. In MEFs, lack of NOXA and BIM conferred significant resistance to fluorizoline-induced apoptosis, thus revealing a key role for these BH3-only proteins in the mechanism of action of fluorizoline. On the other hand, downregulation of both *NOXA* and *BIM* in HeLa cells failed to offer more protection than the absence of *NOXA* alone, thereby suggesting that BIM plays only a minor role in these cells. The consistent upregulation of NOXA protein levels in all the cancer cell types that were analyzed points towards a main function of this BH3-only protein in fluorizoline-induced apoptosis. Compared to PHB-depleted cells, the loss of the crucial BH3-only proteins (i.e. NOXA and BIM for MEFs and NOXA for HeLa cells) blocked fluorizoline-induced apoptosis less efficiently, thus suggesting a contribution of other BCL-2 family members modulated in a PHB-dependent manner.

In addition to their role in mitochondria, PHBs also localize in other cellular compartments, including the nucleus and the plasma membrane, in a wide range of cell types [11, 75]. In the plasma membrane, PHBs have been involved in the activation of the signaling pathways Ras-Raf-MAPK and the tyrosine kinase Syk [76-79]. In the nucleus, PHBs were reported to regulate the activity of various transcription factors such as p53, E2F1, and estrogen receptor [11]. Whether the fluorizoline-induced transcriptional modulations are directly mediated by nuclear PHBs or indirectly mediated by PHBs in other cellular compartments requires further analysis.

Similar to fluorizoline, the effects of three natural products that bind to PHBs have been reported. First, capsaicin binds to PHB2 to induce cytochrome *c* release from mitochondria [80]. Second, the depsipeptide aurilide was shown to selectively bind to PHB1 in the mitochondria, leading to rapid mitochondrial fragmentation and the induction of apoptosis [81]. Third, Rocaglamide A, a member of the flavagline class of compounds, binds to both PHB1 and PHB2 at the cell membrane to inhibit Raf activation and Raf-MEK-ERK-mediated cell cycle progression and cell proliferation in tumor cell lines, without inducing mitochondrial fragmentation [82]. Interestingly, Rocaglamide A and other related flavaglines have pro-apoptotic activities both *in vitro* and *in vivo* in various cancer cells; however, it remains unknown whether PHBs are involved in their mechanism of apoptosis induction [11, 83]. The complexity of the synthesis or the restricted availability of these compounds may hinder their use in therapeutics. In contrast, fluorizoline synthesis is practical, versatile, short, and reproducible, and thus amenable to gram-scale batches [2]. *In vivo*, sub-acute toxicity studies in mice revealed that fluorizoline only produces some liver toxicity at the higher doses that were tested (data not shown). Thus, it would be possible to analyze its anti-tumor effects *in vivo*.

In conclusion, this study demonstrates that PHBs are required for fluorizoline-induced apoptosis. PHBs mediate the upregulation of NOXA and BIM, which are crucial for the pro-apoptotic effects of fluorizoline. Moreover, our findings reveal that PHBs are amenable to modulation by small molecules to finally induce apoptosis, and thus further contributes to highlighting the suitability of PHBs as promising therapeutic targets.

## MATERIALS AND METHODS

### Reagents

The synthesis of racemic fluorizoline was performed as previously described [2]. Actinomycin D was purchased from Enzo Life Sciences (Farmingdale, New York, USA). Q-VD-OPh and Z-IETD-FMK were from R&D systems (Minneapolis, Minnesota, USA). Recombinant mouse TNFα was from PeproTech (Rocky Hill, New Jersey, USA). MnTBAP, etoposide and cycloheximide were from Sigma-Aldrich (Saint Louis, Missouri, USA).

### Cell lines culture

WT and *Phb2^fl/fl^* MEFs were generated as previously described [10]. *Phb2^fl/−^* MEFs were generated by transducing *Phb2^fl/fl^* MEFs with Cre recombinase for 72 h. Cells were sorted individually in a 96-well plate and then allowed to grow for several days. Genotyping was performed by PCR analysis as previously described [10]. MEFs WT, *Bax*^−/−^, *Bak*^−/−^ and *Bax*^−/−^/*Bak*^−/−^ MEFs were obtained from Dr. Korsmeyer's laboratory [50]. HeLa and Jurkat cells were supplied by the European Collection of Cell Culture. *Oma1^−/−^* MEFs were a kind gift of Dr. Lopez-Otín (Instituto Universitario de Oncología, Universidad de Oviedo, Spain). WT, *Bim*^−/−^, *Noxa*^−/−^ and *Bim*^−/−^/*Noxa*^−/−^ MEFs were kindly provided by Dr. Andreas Villunger (Medical University of Innsbruck, Austria). Jurkat-BCL-X_L_ cells were generated by Dr. Victor Yuste (Universitat Autònoma de Barcelona, Spain). *Bax*^−/−^/*Bak*^−/−^ MEFs overexpressing CrmA were kindly provided by Dr. Muñoz-Pinedo [84]. HT29 and MW1552 cells were supplied by ATCC. A375P cells were provided by Dr. I. J. Fidler (MD Anderson Cancer Center, Houston, USA).

All cell lines were cultured in Dulbecco's Modiﬁed Eagle Medium except for Jurkat, which were cultured in Roswell Park Memorial Institute (RPMI) 1640 Medium supplemented with 10% fetal bovine serum, 2 mM L-glutamine, 100 U/mL penicillin, and 100 ng/mL streptomycin (all from Biological Industries, Israel) at 37°C in a humidiﬁed atmosphere containing 5% carbon dioxide.

### Primary samples and cell isolation

Peripheral blood samples and bone marrow aspirates from untreated patients were obtained after informed consent in accordance with protocols approved by the Human Research Ethics Committees of the Hospital ICO-Duran i Reynals, L'Hospitalet de Llobregat, Spain. Patients with chronic myeloid leukemia-derived blast crisis, mantle cell lymphoma, B cell chronic lymphoproliferative syndrome and adult T-cell leukemia/lymphoma were diagnosed according to standard clinical and laboratory criteria. Peripheral blood and bone marrow mononuclear cells were isolated by centrifugation on a Biocoll (Biochrom AG, Berlin, Germany) gradient and cryopreserved in liquid nitrogen in the presence of 10% DMSO (Sigma-Aldrich). Mononuclear cells were cultured immediately after thawing or isolation at a concentration of 1×10^6^ cells/mL in RPMI 1640 culture medium supplemented with 10% heat-inactivated fetal bovine serum, 2 mM L-glutamine, 100 U/mL penicillin, and 100 μg/mL streptomycin (all from Biological Industries) at 37°C in a humidified atmosphere containing 5% carbon dioxide.

### Transduction of MEFs with Cre-recombinase

Recombinant His-TAT-NLS-Cre-recombinase (HTNC) was expressed and purified as previously described [85]. 6 μM HTNC was diluted in DMEM/PBS (1:1), sterile filtered and applied to WT and *Phb2^fl/fl^* MEFs for 20 h. Afterwards, cells were washed with PBS and grown in complete medium for 48 h. MEFs were then treated with fluorizoline for 24 h.

### Analysis of cell viability by flow cytometry

Cell viability was measured by exposure of phosphatidylserine and expressed as the percentage of annexin V-APC-negative population. Cells were washed and incubated with annexin binding buffer and annexin V-APC for 15 min in the dark and then analyzed by flow cytometry using FACSCalibur and CellQuest-Pro software (BD Biosciences, Franklin Lakes, New Jersey, USA).

### Western blot

Whole cell protein extracts were obtained by lysing cells with Laemmli sample buffer or RIPA buffer (Supplementary Figure 1b). Protein concentration was measured with the Micro BCA Protein Assay Reagent kit (Pierce, Rockford, Illinois, USA). 20-50 μg of protein extracts were subjected to reducing conditions, loaded onto a polyacrylamide gel and then transferred to Immobilon-P membranes from Millipore (Billerica, Massachusetts, USA) or Protran 0.2 nitrocellulose membranes from Amersham (Buckinghamshire, UK) (Supplementary Figure 1b). One hour after blocking with 5% (w/v) non-fat milk in Tris-buffered saline with Tween^®^ 20, membranes were incubated with the following specific primary antibodies: cleaved caspase 3 (9661, Cell Signalling, Danvers, Massachusetts, USA); PARP (9542, Cell Signalling); OPA1 (612607, BD Biosciences); PHB1 (H-80, Santa Cruz, Dallas, Texas, USA); PHB2 (07-234, Millipore); Tubulin (CP06, Millipore); β-Actin (A5441, Sigma-Aldrich); ERK2 (clone 1B3B9, 05-157, Millipore); BAX (AB2915, Millipore); BAK (06-536, Millipore); BCL-X_L_ (B22630, Transduction Laboratories); MCL-1 (S-19, sc-819, Santa Cruz); BIM (2933, Cell Signaling); NOXA (ab13654, Abcam, Cambridge, UK); PUMA (4976, Cell Signaling); and BCL-2 (M0887, Dako, Santa Clara, California, USA). For Supplementary Figure 1b, we used different antibodies for PHB1 (603102, BioLegend, San Diego, California) and PHB2 (611802, BioLegend). Antibody binding was detected using a secondary antibody conjugated to horseradish peroxidase, and the enhanced chemiluminescence detection system (Amersham). Molecular size markers (#26619, Thermo Fisher Scientific) are indicated in kDa in each figure.

### Transient transfection

siRNA transfection. MEFs were transfected with scramble (12935-300) or *Phb2* siRNA (MSS236022), while HeLa cells were transfected with scramble (12935-300), *BAK* (HSS141354, HSS141355, HSS141356), *BAX* (HSS184085, HSS184086, HSS184087), *NOXA* (HSS143341, HSS143342, HSS143343), or *BIM* (s195011) siRNA using the Lipofectamine^®^ RNAiMax Reagent (all from Thermo Fisher Scientific, Waltham, Massachussets, USA). Complexes were added directly to growing cells in DMEM and incubated for 24 h, followed by washing with PBS buffer and addition of fresh DMEM. MEFs were used in experiments 72 h after siRNA transfection, and HeLa cells were used after 48 h. The efficiency of the downregulation was assessed by western blot. Plasmid transfection. HeLa cells were transfected with either pcDNA3.1, OPA1-ΔS1 construct (kindly provided by Dr. Ishihara, Kurume University, Japan) or 3X-FLAG PHB1 and PHB2 plasmids (a kind gift of Dr. Handa, Tokyo Institute of Technology, Japan) using the Lipofectamine^®^ LTX Reagent (Thermo Fisher Scientific). Cells were used in experiments 48 h after transfection. MEFs were transfected with PHB1 and PHB2 overexpression plasmids using the Lipofectamine^®^ 2000 Reagent (Thermo Fisher Scientific). Complexes were incubated for 25 min at room temperature and added dropwise to growing cells in DMEM. Cells were used in experiments 16 h after transfection. The efficiency of the overexpression was assessed by western blot.

### Fluorescence imaging

HeLa cells were grown on sterilized poly-L-lysine-coated coverslips (0.01% solution, Sigma-Aldrich) and incubated with 100 nM MitoTracker^®^ Red CMXRos (Thermo Fisher Scientific) for 30 min at 37°C. Cells were visualized using spectral confocal microscope (TCS-SL, Leica Microsystems, Wetzlar, Germany) and a Plan-Apochromat 63×/1.4 N.A. immersion oil objective (Leica Microsystems). Images were acquired using the accompanying image processing software from Cytovision.

### Transmission electron microscopy

HeLa cells were rinsed with PBS and immediately fixed by immersion in 0.1 M glutaraldehyde in phosphate buffer for 2-3 h at 4°C. Cells were gently scraped, collected, and pelleted by centrifugation. Cells were treated with 1% osmium tetroxide and after dehydration were embedded in epoxy resin (Araldite, Basel, Switzerland). Thin sections were cut with a diamond knife on a ultramicrotome (Ultracut, Reichert-Jung, Depew, New York, USA), stained with uranyl actetate/lead citrate and each sample was placed into grids to be examined in a JEOL 1010 electron microscope equipped with a Bioscan digital camera (Gatan, Pleasanton, California, USA).

### ROS production

Superoxide anion and hydroxyl radical production was analyzed by incubating Jurkat or MEF cells with 2.5 μM CellROX^®^ Deep Red Reagent (Thermo Fisher Scientific) during the last 30 min of the treatment at 37°C. ROS production was measured by flow cytometry using FACSCalibur and CellQuest-Pro software (BD Biosciences), and expressed as the mean fluorescence intensity relative to the mean of the control.

### Measurement of mitochondrial membrane potential

Jurkat cells were stained with 100 nM MitoTracker^®^ Red CMXRos (Thermo Fisher Scientific) during the last 30 min of the treatment at 37°C and then analyzed by flow cytometry using MoFlo^®^ Astrios (Beckman Coulter, Brea, California, USA). Decreased fluorescence indicates a loss of ΔΨm.

### Reverse transcriptase multiplex ligation-dependent probe amplification (RT-MLPA)

RNA content was analyzed by RT-MLPA using the SALSA MLPA KIT RM002-B1 (MEFs) and R011-C1 (HeLa cells) Apoptosis mRNA from MRC-Holland (Amsterdam, The Netherlands) for the simultaneous detection of 40 mRNA molecules [86]. In brief, 200 ng total RNA were first reverse transcribed using a gene specific primer mix. The resulting cDNA was annealed overnight at 60°C to the RT-MLPA probe mix. Annealed oligonucleotides were ligated by adding Ligase-65 (MRC-Holland) and incubated at 54°C for 15 min. Ligation products were amplified by PCR (35 cycles, 30 s at 95°C; 30 s at 60°C, and 1 min at 72°C) with one unlabeled and one FAM-labeled primer. PCR fragments were separated by capillary electrophoresis on a 48-capillary ABI-Prism 3730 Genetic Analyzer (Applied Biosystems, California, USA). Peak area and height were measured using GeneMapper v3.0 analysis software (Applied Biosystems). Individual peaks were calculated relative to the sum of all peak data to normalize for fluctuations in total signals between samples. Data are shown as the mean ± SEM relative to the mean of the controls.

### Statistical analysis

The results are shown as the mean ± standard error of the mean (SEM) of values obtained in three or more independent experiments. Statistical analysis was performed using the Student's t test (two-tailed) by using GraphPad Prism 6.0c Software Inc. *p* values below 0.05 were considered statistically significant (*p < 0.05; **p < 0.01; ***p < 0.001).

## SUPPLEMENTARY MATERIAL FIGURES


