# Supplementary material for: A novel prohibitin-binding compound induces the mitochondrial apoptotic pathway through NOXA and BIM upregulation
Source: Oncotarget. 2015 Oct 19;6(39):41750–65. doi: 10.18632/oncotarget.6154 (PMC4747186; doi:10.18632/oncotarget.6154)
Supplement: Supplementary file 1 [file oncotarget-06-41750-s001.pdf]

A novel prohibitin-binding compound induces the mitochondrial apoptotic pathway through NOXA and BIM upregulation

Supplementary Material

Supplementary Figure 1

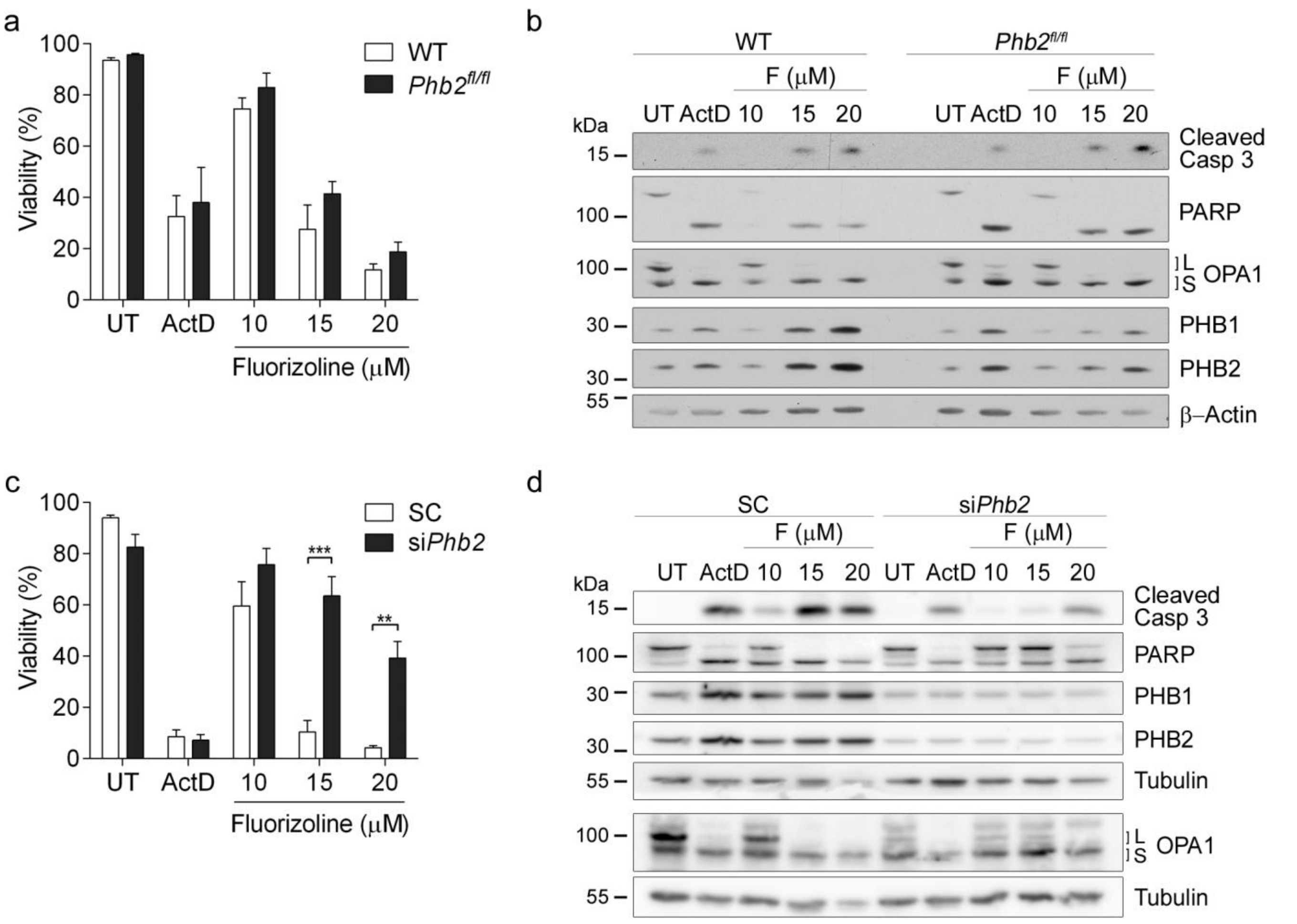

Supplementary Figure 1. The presence of PHBs is required for fluorizoline-induced apoptosis. (a, b) WT and *Phb2*$^{fl/fl}$ MEFs were untreated (UT) or treated with either 0.15 µg/mL Actinomycin D (ActD) or increasing doses of fluorizoline (F) for 24 h. **(c, d)** WT MEFs were transfected with scramble (SC) or *Phb2* siRNA (si*Phb2*) for 72 h. Afterwards, cells were treated with either 2.5 µg/mL Actinomycin D (ActD) or increasing doses of fluorizoline (F) for 24 h. **(a, c)** Viability was measured by flow cytometry and it is expressed as the mean ± SEM (n≥3) of the percentage of non-apoptotic cells (annexin V-negative). $^{**}p < 0.01$, $^{***}p < 0.001$. **(b, d)** Protein levels were analyzed by western blot. β-Actin and Tubulin were used as a loading control. These are representative images of at least three independent experiments.

Supplementary Figure 2

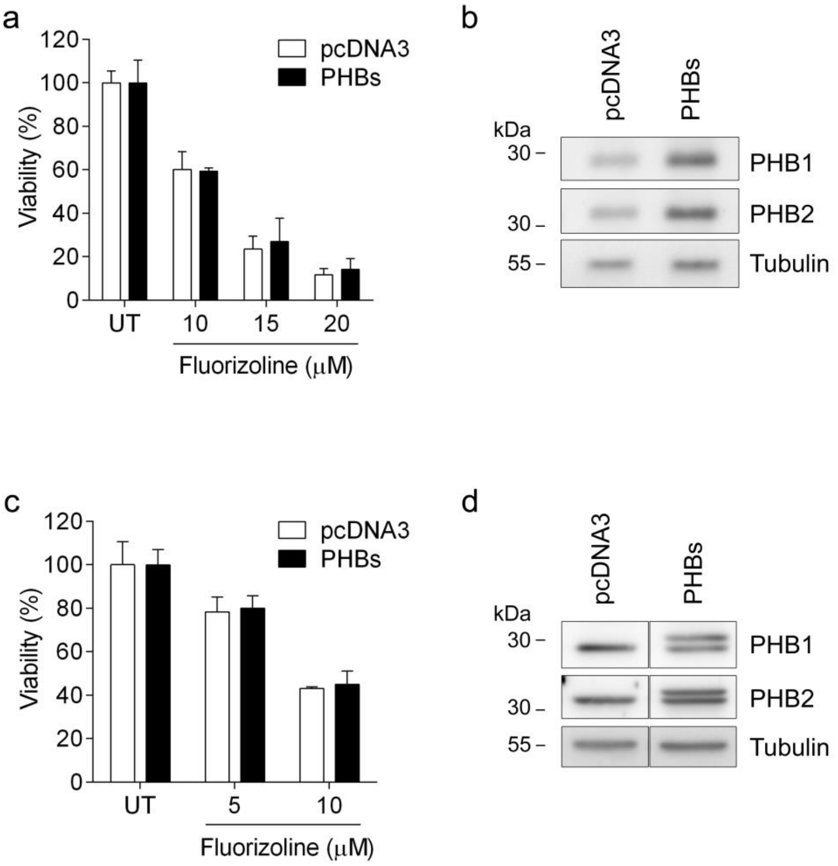

Supplementary Figure 2. Overexpression of PHBs does not render cells resistant to fluorizoline treatment. (a, b) *Phb2*$^{fl/fl}$ MEFs were transfected with pcDNA3.1 vector or with PHB1 and PHB2 expression vectors for 16 h. **(c, d)** HeLa cells were transfected with pcDNA3.1 vector, or 3xFLAG-PHB1 and 3xFLAG-PHB2 expression vectors for 24 h. **(a, c)** Cells were then untreated (UT) or treated with increasing doses of fluorizoline for 24 h. Viability was measured by flow cytometry and it is expressed as the mean ± SEM (n=3) of the percentage of non-apoptotic cells (annexin V-negative) relative to the mean of the controls. **(b, d)** After 24 h of transfection, cells were collected and protein levels were analyzed by western blot. Tubulin was used as a loading control. These are representative images of three independent experiments. In d, the upper band in PHB1 and PHB2 blots corresponds to 3X-FLAG-PHB1 and 3X-FLAG-PHB2, respectively.

Supplementary Figure 3

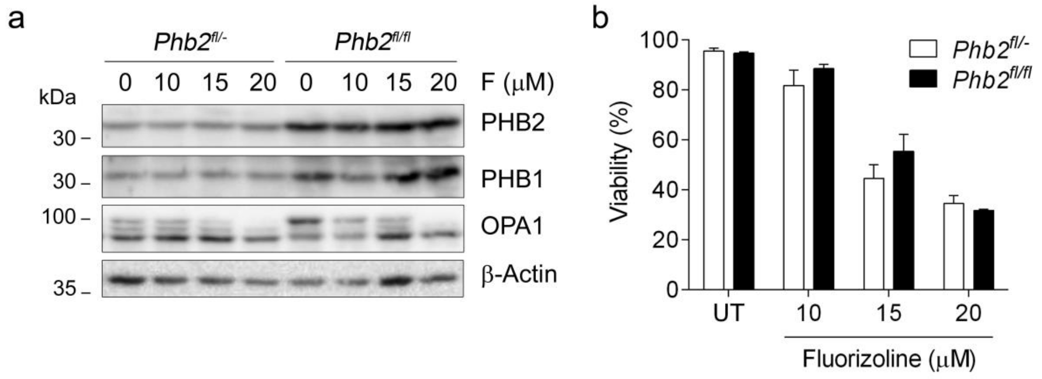

Supplementary Figure 3. *Phb2$^{fl/-}$* and *Phb2$^{fl/fl}$* clones respond similarly to fluorizoline treatment. *Phb2$^{fl/-}$* and *Phb2$^{fl/fl}$* clones were untreated (UT) or treated with 10, 15 or 20 µM fluorizoline (F) for 24 h. **(a)** Protein levels were analyzed by western blot. β-Actin was used as a loading control. This is a representative image of two independent experiments. **(b)** Viability was measured by flow cytometry and it is expressed as the mean ± SEM (n=2) of the percentage of non-apoptotic cells (annexin V-negative).

Supplementary Figure 4

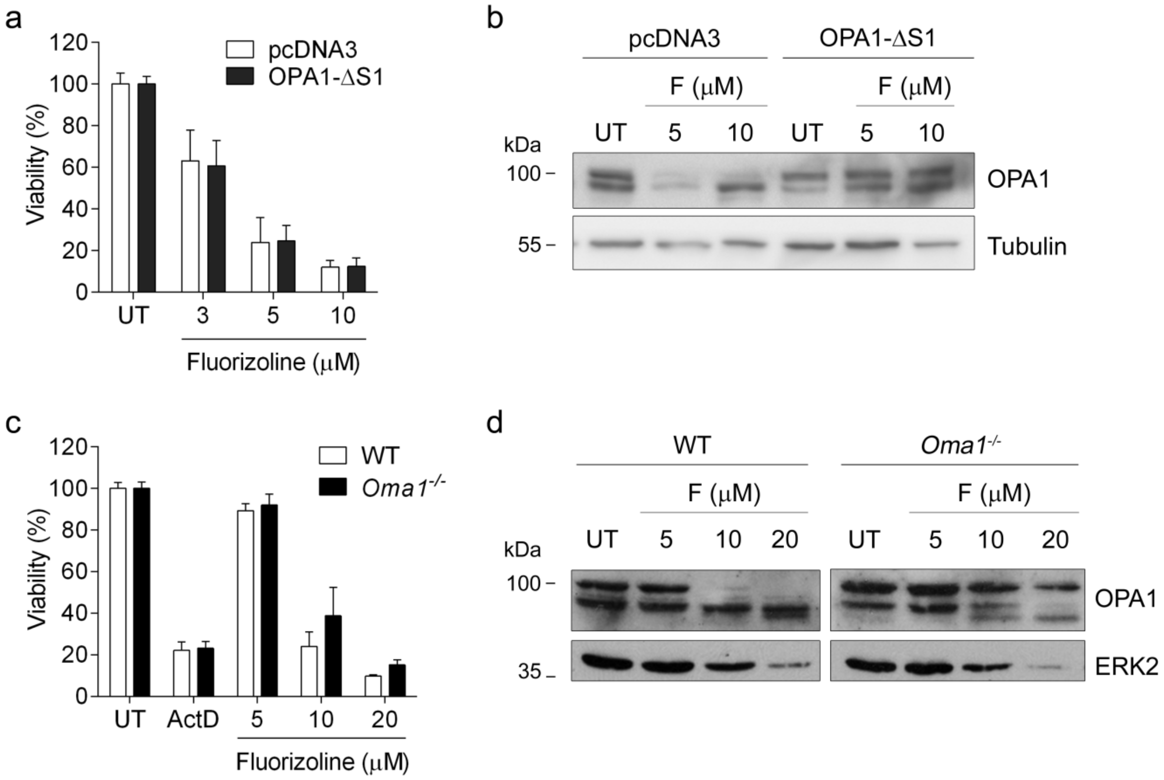

Supplementary Figure 4. Fluorizoline-induced apoptosis is independent of the presence of L-OPA1. (a, b) HeLa cells were transfected with pcDNA3.1 vector or OPA1-ΔS1 expression vector for 48 h. Afterwards, cells were untreated (UT) or treated with increasing doses of fluorizoline (F) for 24 h. **(c, d)** WT and *Oma1*$^{-/-}$ MEFs were untreated (UT) or treated with either 2.5 μg/mL Actinomycin D (ActD) or increasing doses of fluorizoline (F) for 24 h. **(a, c)** Viability was measured by flow cytometry and it is expressed as the mean ± SEM (n=4) of the percentage of non-apoptotic cells (annexin V-negative) relative to the mean of the controls. **(b, d)** Protein levels were analyzed by western blot. In b, OPA1-ΔS1 and endogenous L- and S-forms of OPA1 are detected. Tubulin and ERK2 were used as a loading control. These are representative images of four independent experiments.

Supplementary Figure 5

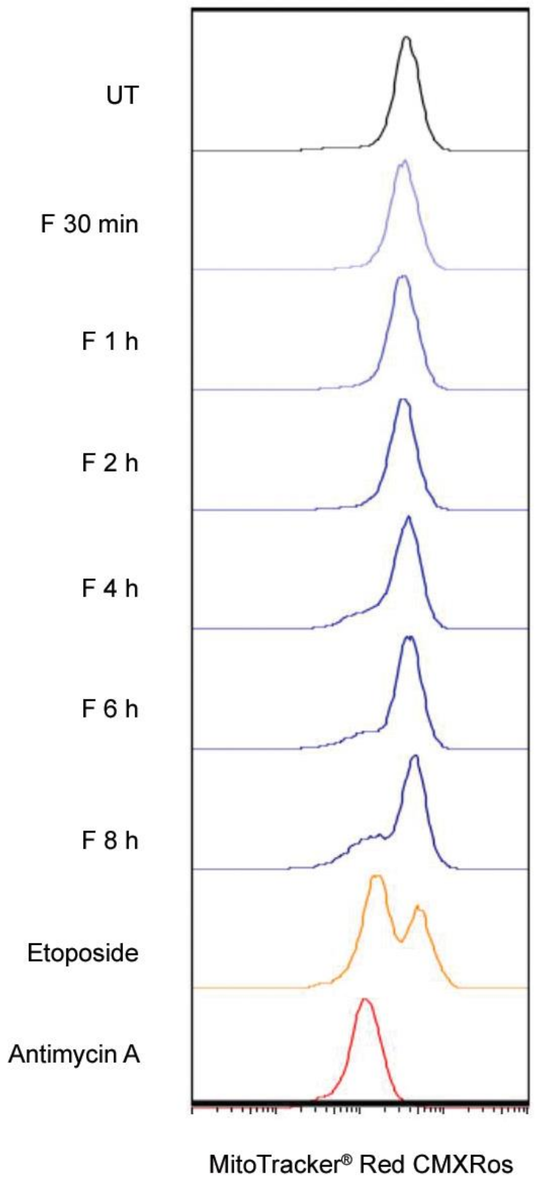

Supplementary Figure 5. Analysis of the mitochondrial membrane potential of Jurkat cells. Jurkat cells were untreated (UT), or treated with 10 µM fluorizoline (F) for different times, 12.5 µM etoposide, a cell death inducer, for 8 h or 200 µM antimycin A, an inhibitor of complex III of the respiratory chain, for 30 min. Cells were stained with 100 nM MitoTracker® Red CMXRos for 30 min at 37ºC and then analyzed by flow cytometry. Decreased fluorescence indicates a loss of $\Delta\Psi m$. These are representative histograms of the fluorescence intensity of MitoTracker® Red CMXRos of three independent experiments.

Supplementary Figure 6

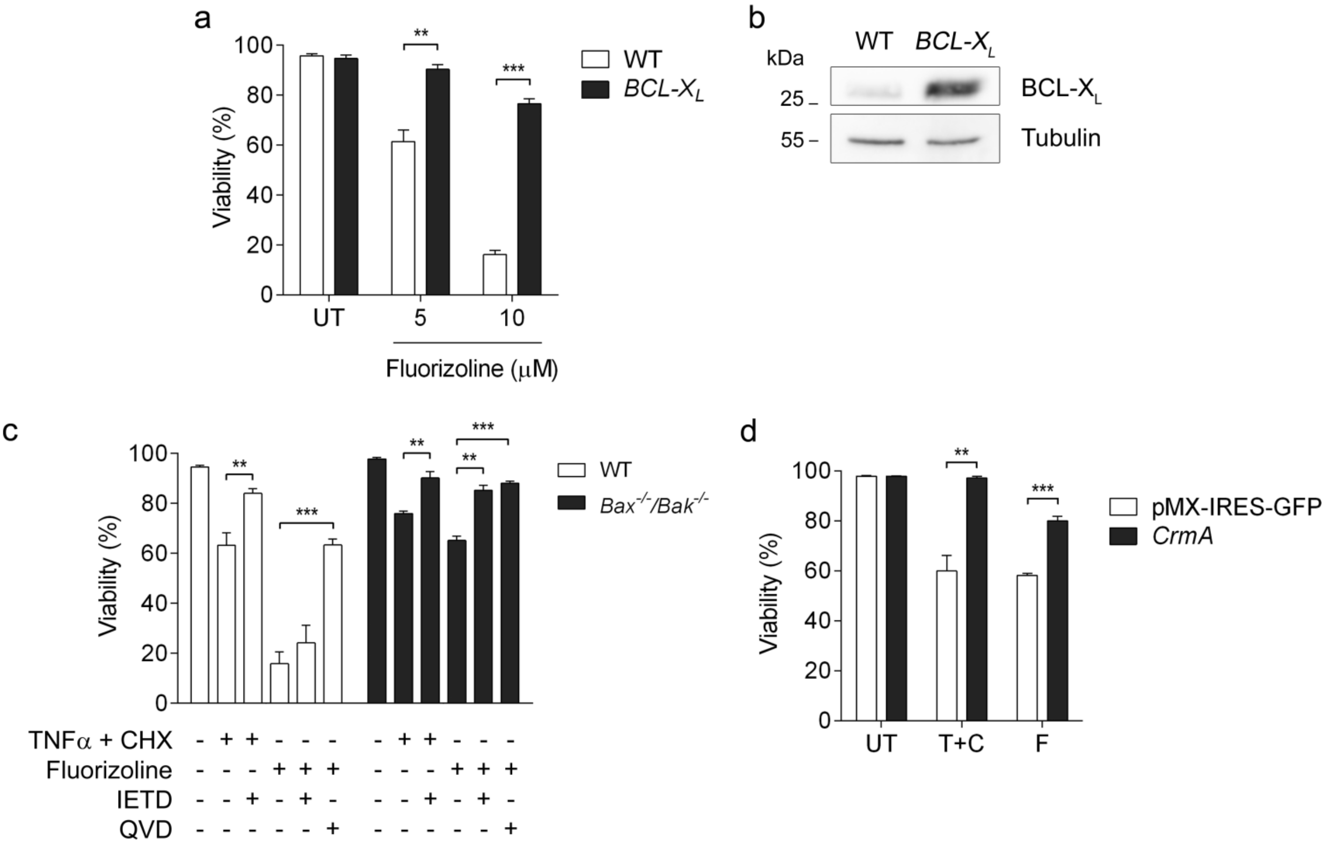

Supplementary Figure 6. Characterization of fluorizoline-induced apoptosis. (a, b) WT Jurkat cells or Jurkat cells overexpressing BCL-X_L were untreated (UT) or treated with different doses of fluorizoline for 24 h. **(c)** WT and *Bax*$^{-/-}$/*Bak*$^{-/-}$ MEFs were pre-incubated or not with Z-IETD-FMK or Q-VD-OPh for 1 h and then treated either with fluorizoline or a combination of TNFα and cycloheximide (TNFα+CHX) for 24 h (for WT MEFs, 50 μM z-IETD-FMK, 20 μM Q-VD-OPh, 20 μM fluorizoline, 10 ng/mL TNFα and 0.5 μg/mL CHX; for *Bax*$^{-/-}$/*Bak*$^{-/-}$ MEFs, 25 μM Z-IETD-FMK, 20 μM Q-VD-OPh, 20 μM fluorizoline, 30 ng/mL TNFα and 2 μg/mL CHX). **(d)** *Bax*$^{-/-}$/*Bak*$^{-/-}$ MEFs stably transfected with pMX-IRES-GFP or CrmA expression vector were untreated (UT) or treated with 30 ng/mL TNFα and 2 μg/mL cycloheximide (T+C) or 20 μM fluorizoline (F) for 24 h. **(a, c, d)** Viability was measured by flow cytometry and it is expressed as the mean ± SEM (n≥3) of the percentage of non-apoptotic cells (annexin V-negative). **(b)** Protein levels were analyzed by western blot. Tubulin was used as a loading control. This is a representative image of three independent experiments.

Supplementary Figure 7

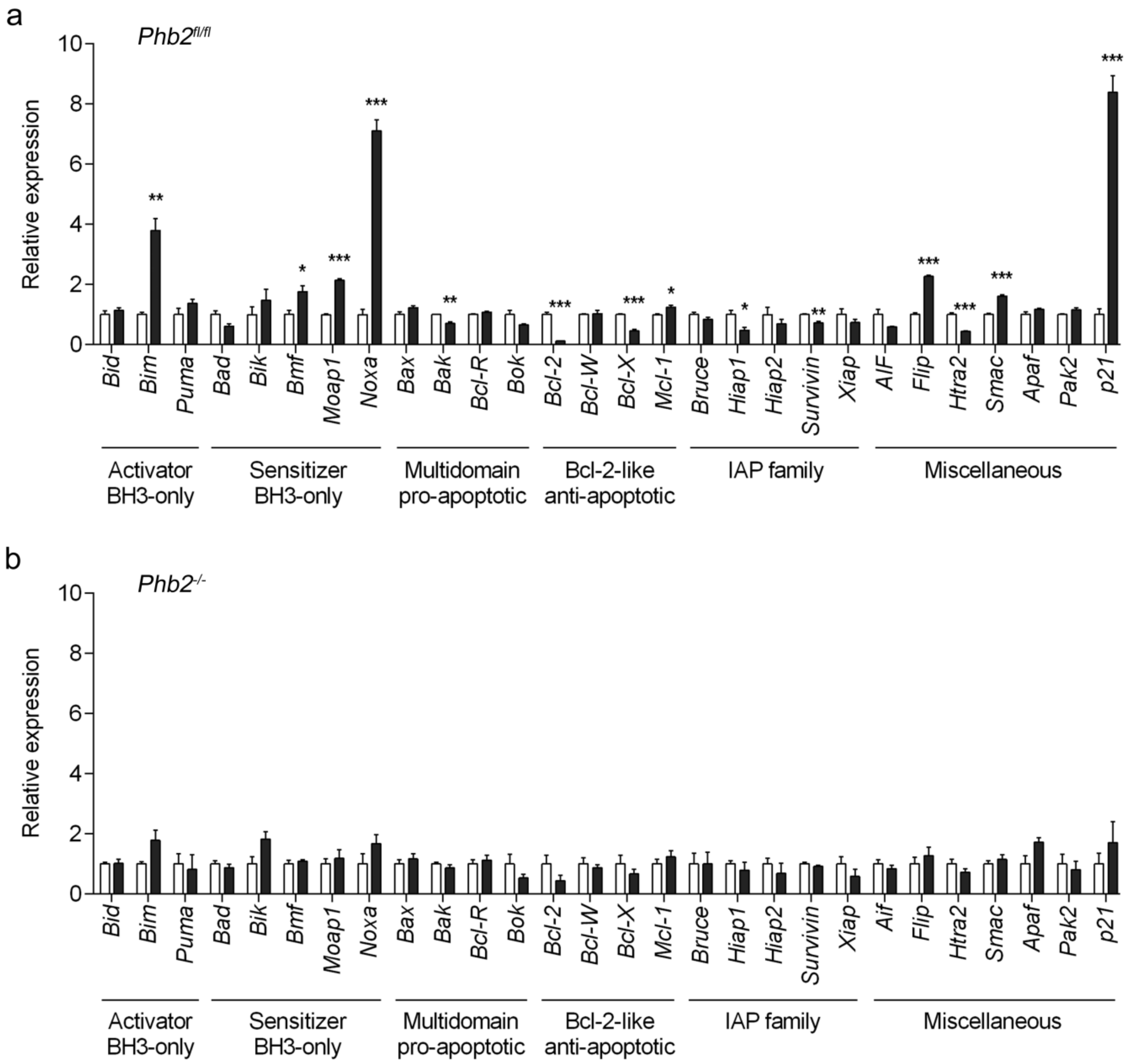

Supplementary Figure 7. RT-MLPA analysis show PHB-dependent modulation of apoptosis-related gene expression profile upon fluorizoline treatment in *Phb2$^{fl/fl}$* MEFs. (a) *Phb2$^{fl/fl}$* MEFs were untreated (white bars) or treated with 20 µM fluorizoline (black bars) for 24 h. **(b)** *Phb2$^{fl/fl}$* MEFs were transduced with Cre recombinase for 72 h (*Phb2$^{-/-}$*) and then untreated or treated with 20 µM fluorizoline for 24 h. **(a, b)** mRNA levels were analyzed by RT-MLPA. White bars correspond to untreated cells, and black bars to fluorizoline-treated cells. Data show the mean values ± SEM of three independent experiments relative to the mean of the control. *$p < 0.05$, **$p < 0.01$, ***$p < 0.001$ untreated versus treated cells.

Supplementary Figure 8

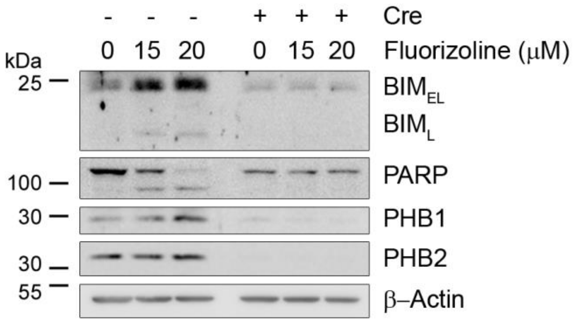

Supplementary Figure 8. PHBs are required for fluorizoline-induced increases in BIM protein levels. *Phb2*$^{fl/fl}$ MEFs were transduced or not with Cre recombinase for 72 h and then untreated or treated with 15 or 20 µM fluorizoline for 24 h. Protein levels were analyzed by western blot. β-Actin was used as a loading control. This is a representative image of at least three independent experiments.

Supplementary Figure 9

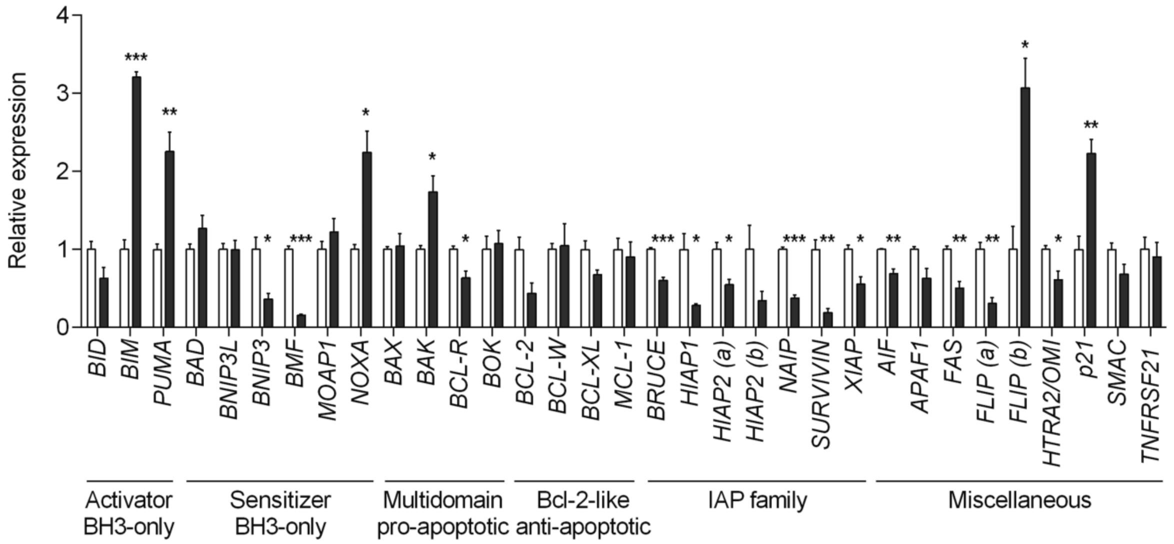

Supplementary Figure 9. Fluorizoline-induced modulation of the apoptosis-related gene expression profile in HeLa cells. HeLa cells were untreated (white bars) or treated with 10 µM fluorizoline (black bars) for 24 h. mRNA levels were analyzed by RT-MLPA. Data show the mean values ± SEM of three independent experiments relative to the mean of the control. *$p < 0.05$, **$p < 0.01$, ***$p < 0.001$ untreated versus treated cells.

Supplementary Figure 10

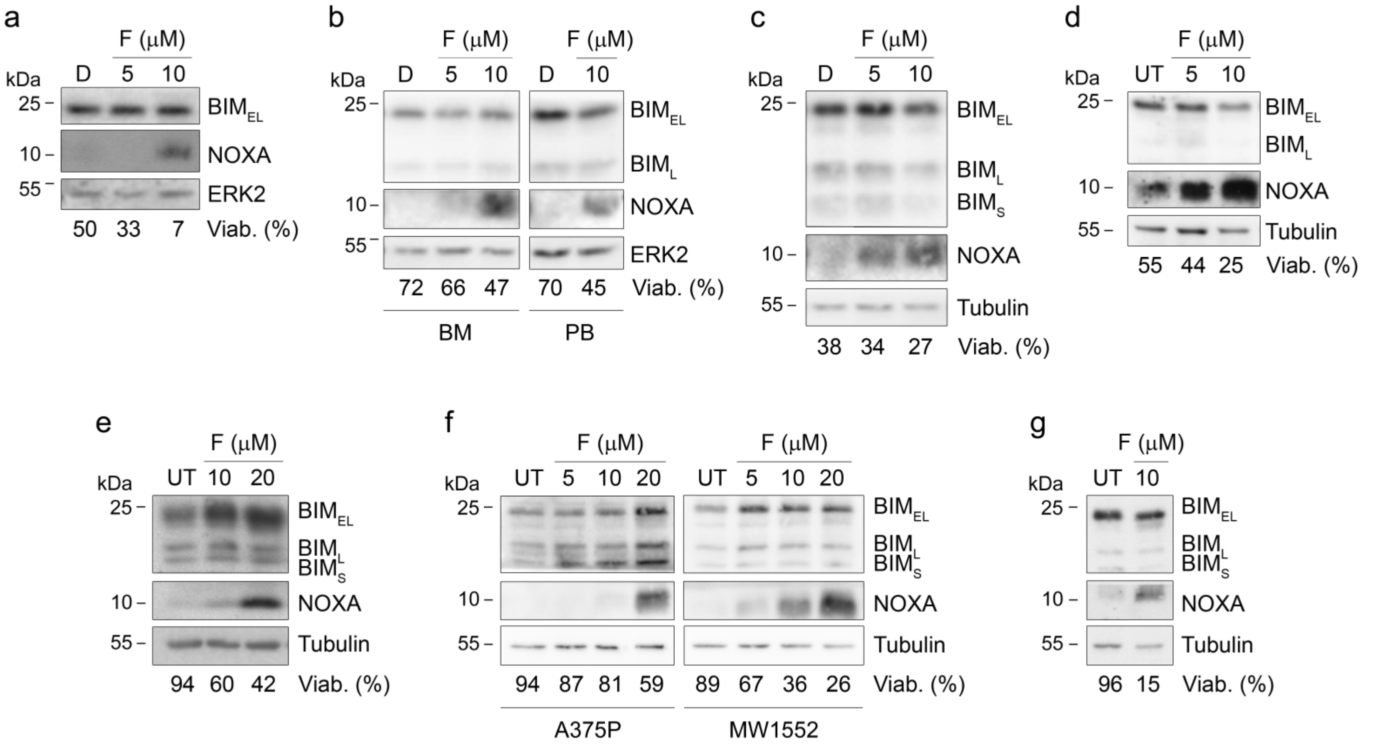

Supplementary Figure 10. Analysis of BIM and NOXA protein levels in different cancer cell types. Primary cells from chronic myeloid leukemia-derived blast crisis **(a)**, from mantle cell lymphoma (bone marrow aspirate –BM-, or a peripheral blood sample –PB-) **(b)**, from B cell chronic lymphoproliferative syndrome **(c)**, and from adult T-cell leukemia/lymphoma **(d)**, as well as the human colon cancer cell line HT29 **(e)**, the melanoma cell lines A375P and WM1552 **(f)**, and the acute T cell leukemia cell line Jurkat **(g)** were untreated (UT), or treated with DMSO (D) or increasing doses of fluorizoline (F) for 24 h. BIM and NOXA protein levels were analyzed by western blot. Tubulin or ERK2 were used as loading control. Viability was measured by flow cytometry and it is expressed as the percentage of non-apoptotic cells (annexin V-negative).
